# P-160. Exploring Invasive Fungal Infection Diagnosis and Treatment: A Cross-Continental Comparison between Brazil and Europe

**DOI:** 10.1093/ofid/ofae631.365

**Published:** 2025-01-29

**Authors:** Jon Salmanton-Garcia, Diego R Falci, Oliver A Cornely, Alessandro Pasqualotto

**Affiliations:** University Hospital Cologne, Cologne, Germany, Cologne, Nordrhein-Westfalen, Germany; PUCRS, Porto Alegre, Rio Grande do Sul, Brazil; University of Cologne, Faculty of Medicine and University Hospital Cologne, Cologne, Nordrhein-Westfalen, Germany; Universidade Federal de Ciências da Saúde de Porto Alegre, Port, Rio Grande do Sul, Brazil

## Abstract

**Background:**

Brazil's tropical climate and a higher prevalence of immunocompromised cases due to conditions like HIV/AIDS contribute to an increased incidence of invasive fungal infections (IFIs) like histoplasmosis and paracoccidioidomycosis. In Europe, vulnerable populations undergoing immunosuppressive therapies or organ transplants face elevated risks of IFIs such as candidiasis and aspergillosis.

**Methods:**

An online survey, conducted to assess diagnostic capacities for IFIs, engaged mycologists across Europe and Brazil. The survey encompassed various aspects, including institutional characteristics, perspectives on IFIs, and the utilization of diagnostic techniques such as microscopy, culture, serology, molecular testing, and therapeutic drug monitoring.

**Results:**

As of 2023, the survey encompassed 484 centers, with 96 in Brazil and 388 in Europe. In Brazil, 45% of centers had on-site mycological diagnostics, while in Europe, this figure was higher at 58% (p< 0.001). Breaking down diagnostic test availability in Brazil: microscopy was accessible in 96% of centers, culture in 95%, antigen detection in 63%, antibody detection in 47%, and molecular tests in 19%. In contrast, in Europe, the distribution was: culture in 99% of centers, microscopy in 97%, antigen detection in 94%, molecular tests in 85%, and antibody detection in 82%. Significant statistical disparities were observed in antigen detection, molecular tests, and antibody detection (p< 0.001). Concerning antifungal treatment in Brazil, at least one formulation of amphotericin B was used in 72% of centers, at least one echinocandin in 55%, fluconazole in 93%, and the primary mould-active azole, itraconazole, in 66%. In Europe, at least one formulation of amphotericin B was used in 87% of centers, at least one echinocandin in 89%, fluconazole in 93%, and the main mould-active azole, itraconazole, in 89%. A notable difference was observed in access to all antifungals (p< 0.001).

**Conclusion:**

Brazil's healthcare disparities and limited access to advanced care impact the diagnosis and management of IFIs, while Europe's widely-established healthcare infrastructure facilitates early detection and treatment. A call to action for the improvement of capabilities in Brazil is warranted.

**Disclosures:**

**Oliver A. Cornely, Prof. Dr.**, Abbott: Honoraria|Abbvie: Advisor/Consultant|Abbvie: Honoraria|AiCuris: Advisor/Consultant|Akademie fur Infektionmedizin: Honoraria|Al-Jazeera Pharmaceuticals/Hikma: Honoraria|amedes: Honoraria|AstraZeneca: Honoraria|Basilea: Advisor/Consultant|Biocon: Advisor/Consultant|BMBF: Grant/Research Support|Boston Strategic Partners: Advisor/Consultant|CIdara: Advisor/Consultant|CIdara: Expert Testimony|CIdara: Grant/Research Support|CIdara: Participation on a DRC or DSMB|CoRe Consulting: Stocks/Bonds (Private Company)|Deutscher Arzteverlag: Honoraria|DZIF: Grant/Research Support|EasyRadiology: Stocks/Bonds (Private Company)|EU-DG RTD: Grant/Research Support|F2G: Grant/Research Support|Gilead: Advisor/Consultant|Gilead: Grant/Research Support|Gilead: Honoraria|Grupo Biotoscana/United Medical/Knight: Honoraria|GSK: Advisor/Consultant|GSK: Honoraria|IQVIA: Advisor/Consultant|IQVIA: Participation on a DRC or DSMB|Janssen: Advisor/Consultant|Janssen: Participation on a DRC or DSMB|Matinas: Advisor/Consultant|MedPace: Advisor/Consultant|MedPace: Grant/Research Support|MedPace: Participation on a DRC or DSMB|Medscape/WebMD: Honoraria|MedUpdate: Honoraria|Menarini: Advisor/Consultant|Moderna: Honoraria|Molecular Partners: Advisor/Consultant|MSD: Grant/Research Support|MSD: Honoraria|MSG-ERC: Advisor/Consultant|Mundipharma: Advisor/Consultant|Mundipharma: Grant/Research Support|Mundipharma: Honoraria|Noscendo: Honoraria|Noxxon: Advisor/Consultant|Octapharma: Advisor/Consultant|Octapharma: Grant/Research Support|Pardes: Advisor/Consultant|Partner Therapeutics: Advisor/Consultant|Patent: US18/562644|Paul-Martini-Stiftung: Honoraria|Pfizer: Advisor/Consultant|Pfizer: Grant/Research Support|Pfizer: Honoraria|PSI: Advisor/Consultant|PSI: Participation on a DRC or DSMB|Pulmocide: Participation on a DRC or DSMB|Sandoz: Honoraria|Scynexis: Advisor/Consultant|Scynexis: Grant/Research Support|Seqirus: Advisor/Consultant|Seqirus: Honoraria|Seres: Advisor/Consultant|Shionogi: Advisor/Consultant|Shionogi: Honoraria|streamedup!: Honoraria|The Prime Meridian Group: Advisor/Consultant|Touch Independent: Honoraria|Vitis: Honoraria

